# Supplementary material for: Effect of 2H and 18O water isotopes in kinesin-1 gliding assay
Source: PeerJ. 2014 Mar 25;2:e284. doi: 10.7717/peerj.284 (PMC3970804; doi:10.7717/peerj.284)

**Step 1:** Microscope slides were centered on the template as shown below.


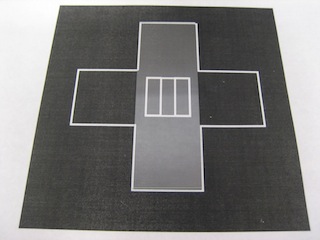


**Step 2:** Two strips of permanent double stick tape (Scotch) were attached to the slide using the two most center lines as a guide. This created a channel that was approximately 10μL in volume. The width of the two center lines was 5mm.


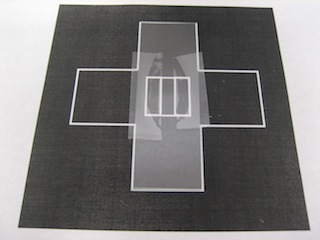


**Step 3:** Excess tape was trimmed by using the outer lines of the center box as a guide. After trimming the tape, a cover slip was placed over the center box.


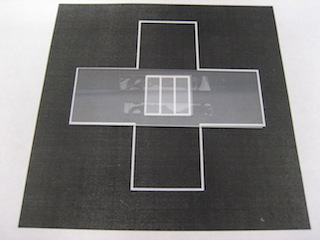


**Step 4:** Adhesion of the cover slip to the tape was ensured by pressing the slip to the slide using a rounded blunt object. Proper adhesion occurred when the light that was scattered from the slide + tape + slip combo differed from when the slip was merely placed on the tape. Compare the figure from step 3 to step 4.


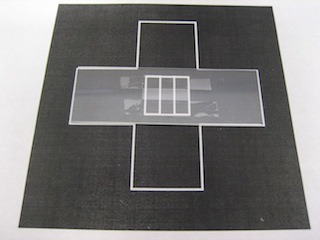


**Step 5:** Two very thin pieces of double stick tape were placed at the entrances of the channel in the flow cell. They have been colored red to enhance contrast. These pieces of tape were essential for proper sealing with a piece of cellophane and helped prevent the objective oil from seeping into the flow cell. The tape strips were made by holding two razor blades together and cutting a thin piece of tape. Excessive evaporation occurred if the strips of tape were not close to the edges of the chamber entrance.


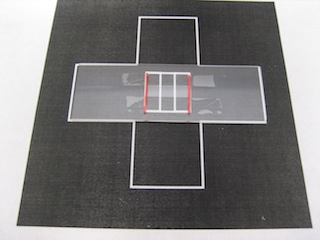


**Step 6:** Small strips of cellophane (Glad Cling Wrap) were then placed over the flow cells and wrapped around the slide. A proper seal ensured that the tape strips adhered to the cellophane.


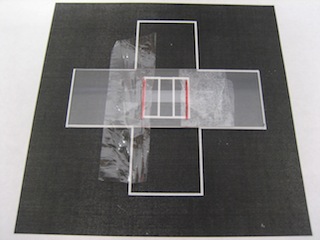

Supplement: Text S1 [file peerj-02-284-s001.docx]
