# Supplementary material for: Effect of 2H and 18O water isotopes in kinesin-1 gliding assay
Source: PeerJ. 2014 Mar 25;2:e284. doi: 10.7717/peerj.284 (PMC3970804; doi:10.7717/peerj.284)
Supplement: Text S2 [file peerj-02-284-s002.docx]

**Resealable flow cell speed decrease.**


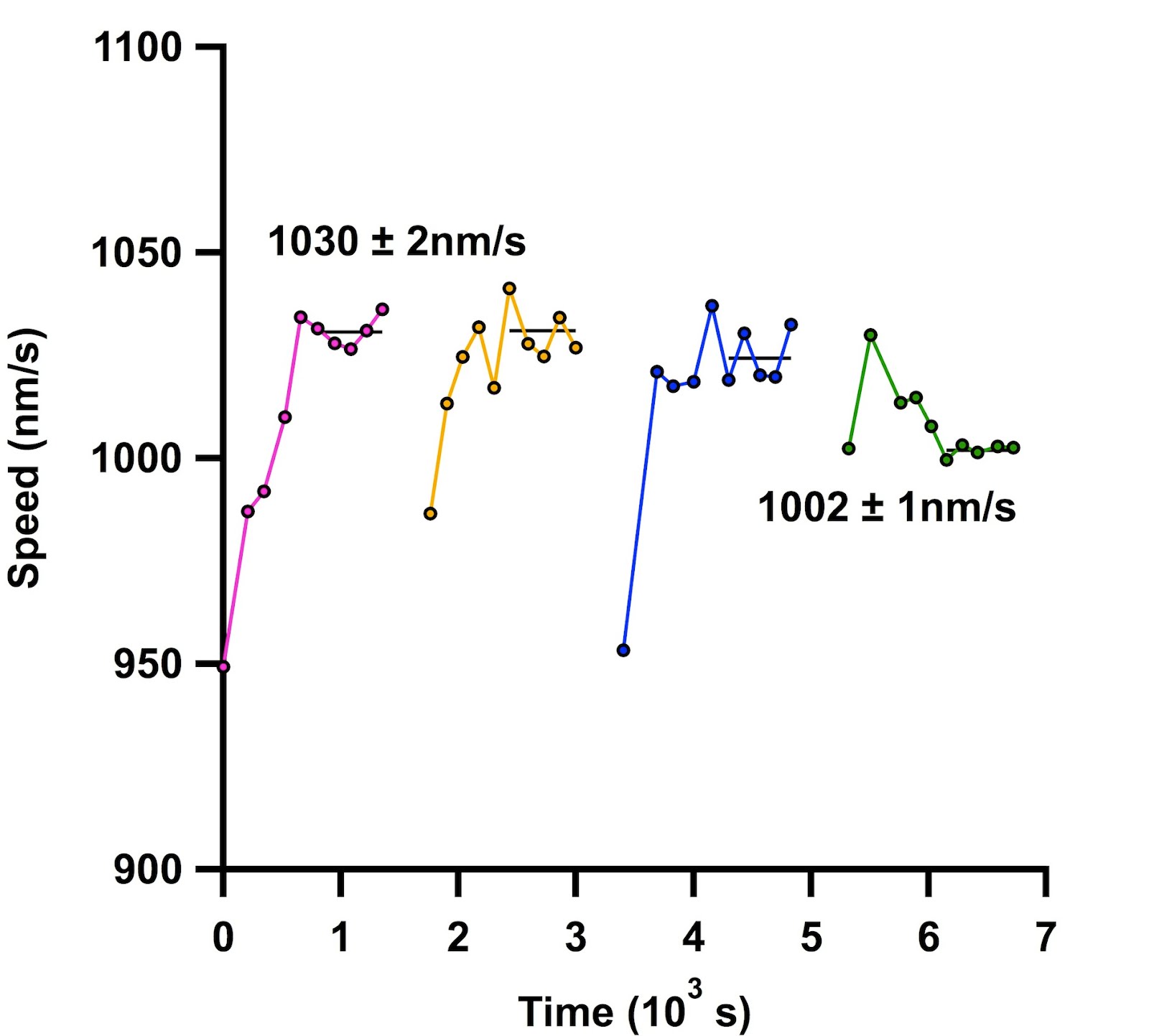


The magenta line has had zero fluid exchanges in the resealable flow cell. Each set of data points further along in time indicate another fluid exchange. The green line (fourth fluid exchange) has a significant decrease in speed measurements as compared to the data with no fluid exchanges. A fifth fluid exchange was done with this flow cell, however, microtubules were untrackable.
